# Oxidative Stress and Autophagy in Metabolism and Longevity

**DOI:** 10.1155/2017/3451528

**Published:** 2017-02-28

**Authors:** Ajit Vikram, Ramakrishnan Anish, Ashutosh Kumar, Durga Nand Tripathi, Ravinder K. Kaundal

**Affiliations:** ^1^Department of Internal Medicine, Carver College of Medicine, University of Iowa, Iowa City, IA 52246, USA; ^2^Department of Biochemistry and Molecular Biology, Baylor College of Medicine, Houston, TX 77030, USA; ^3^Department of Pharmacology and Toxicology, National Institute of Pharmaceutical Education and Research (NIPER), Hyderabad, Telangana 500037, India; ^4^Center for Precision Environmental Health, Department of Molecular and Cellular Biology, Baylor College of Medicine, Houston, TX 77030, USA; ^5^Department of Immunobiology, School of Medicine, Yale University, New Haven, CT 06520, USA

The guest editors of this issue are delighted to present this compendium of research articles focusing on the role of oxidative stress and autophagy in metabolism and longevity. The tilt of oxidant/antioxidant balance in favor of production of free radicals and inability of the cellular antioxidant system to detoxify them constitutes oxidative stress, which plays a crucial role in metabolic disorder and ageing. The connection between reactive oxygen species (ROS) and senescence is widely acknowledged and has formed the mechanistic basis to our understanding for the advancement of ageing. In the recent past the role of ROS as a signaling molecule with specific attributes such as feedback regulation and target selectivity is identified. Hydrogen peroxide, a ROS, offers specific signaling advantages as it has atomic targets instead of conventional molecular targets and therefore can initiate multiple, but yet specific, signaling cascades. A basal level of ROS is essential for the cell signaling and survival, but their elevated levels have been implicated in multiple disease pathologies and ageing. Although existing evidence continues to support the role of oxidative stress in the regulation of metabolism and longevity, the quest to identify therapeutically attractive molecular targets still continues.

ROS and mild chronic inflammation are believed to play a role in the pathogenesis of Parkinson's disease [1, 2], most common age related neurodegenerative disease. In one of the research articles published in this special issue entitled “Altered Striatocerebellar Metabolism and Systemic Inflammation in Parkinson's Disease” authors report the identification of an abnormal level of inflammatory markers in peripheral circulation, as measured by biochemical assays, and in the basal ganglion and cerebellum, as measured by magnetic resonance spectrometry. The study demonstrates the suitability of magnetic resonance spectrometry findings in the brain combined with inflammatory markers in peripheral blood as a new noninvasive means for the early detection of Parkinson's disease.

Although until now it was believed that the dietary habit of mother influences the gene regulation in offspring, it was recently revealed that the dietary habit of father could also influence the future disease susceptibility of daughters [3]. Findings of the research article published in this issue entitled “Parental High-Fat Diet Promotes Inflammatory and Senescence-Related Changes in Prostate” suggest that consumption of fat-rich diet promotes accumulation of p53 expressing cells and proliferation of epithelial cells and senescence-related changes in the prostate. Further, the parental consumption of fat-rich diet was found to uphold the inflammatory, proliferative, and senescence-related changes in prostate of adult pups. The study provides space to debate the dietary habit of parents on the disease susceptibility of offspring and supports the view that the decreased consumption of fat can be a gift to the offspring.

Autophagy is a conserved dynamic catabolic process, in which nonfunctional macromolecules and cellular organelles are engulfed, degraded, and recycled, thereby playing a crucial role in maintaining cellular homeostasis. There are three main types of autophagy: macroautophagy, microautophagy, and chaperone mediated autophagy. During macroautophagy cytoplasmic contents are sequestered in double membrane bound vesicles called autophagosomes and delivered to lysosomes for degradation [4, 5]. In microautophagy, lysosomes engulf the cytoplasmic material by inward invagination of the lysosomal membrane, while chaperone mediated autophagy is facilitated by HSP70, cochaperones, and the lysosomal-associated membrane protein type-2A. Excessive free radicals generated during oxidative stress irreversibly oxidize DNA and cellular biomolecules, thereby representing the primary source of damage in biological systems. Autophagy primarily contributes to clearing the cells of all irreversibly oxidized biomolecules (proteins, DNA, and lipids) and is induced by oxidative stress. Various signaling pathway has been discovered for the induction of autophagy and mTORC1 plays a significant role [6]. However, in some instances autophagy is mTORC1 independent. Autophagy regulates lipid accumulation and adipocyte differentiation and helps to better cope with the starvation stress, all of which have been individually linked to metabolism and longevity. Despite significant development in the understanding of how autophagy regulates cellular process and is being regulated by various factors, its role in metabolism and longevity is not completely elucidated. The mechanistic study by J. Zhou et al., entitled “Thioredoxin Binding Protein-2 Regulates Autophagy of Human Lens Epithelial Cells under Oxidative Stress via Inhibition of Akt Phosphorylation,” describes the importance of autophagy in cataract development via perturbation of thioredoxin system. Thioredoxin binding protein-2 (TBP-2) is a negative regulator of thioredoxin (Trx), which regulates autophagy in the initiation stage under oxidative stress condition and this regulation is mTORC1-independent. Oxidative stress can cause cell injury and autophagy in human lens epithelial cells (LECs), and TBP-2 regulates this response. This study provides evidence regarding the role of TBP-2 in the cataract development. Another interesting study entitled “PTEN Activation by DNA Damage Induces Protective Autophagy in Response to Cucurbitacin B in Hepatocellular Carcinoma Cells” reports that cucurbitacin B induced DNA damage, apoptosis, and protective autophagy are mediated by ROS and PTEN activation in response to DNA damage. These observations provide insights for the better understanding of the crosstalk between ROS and autophagy.

In this special issue a research article entitled “Coordinated Upregulation of Mitochondrial Biogenesis and Autophagy in Breast Cancer Cells: The Role of Dynamin Related Protein-1 and Implication for Breast Cancer Treatment” reports that inhibition of Drp1 significantly suppressed mitochondrial autophagy, metabolic reprogramming, and cancer viability. As basal mitochondrial function is required for cancer cell survival this study supports the view that targeting both mitochondrial biogenesis and turnover could be an attractive strategy in tackling cancer growth. Mitochondria are one of the primary sites of active ROS production. In a proteomics based study by J. Hinkelbein et al., entitled “Decreased Tissue COX5B Expression and Mitochondrial Dysfunction During Sepsis-Induced Kidney Injury in Rats,” authors identify COX5B, a protein involved in the energy production in mitochondria, as a differentially regulated protein in the late phase of the experimental kidney sepsis. They also claim suitability of COX5B as a potential biomarker to identify sepsis; however, they emphasize the need of validation of findings in both human urine and serum.

Circadian Locomotor Output Cycle Protein Kaput (CLOCK) expression follows the circadian-rhythm pattern and is a transcription factor. Endothelial dysfunction precedes and is involved in the pathogenesis of vascular wall disorders. In an interesting mechanistic study entitled “CLOCK Promotes Endothelial Damage by Inducing Autophagy through Reactive Oxygen Species” authors try to establish a connection between ROS, autophagy, and endothelial dysfunction. Overexpression or inhibition of CLOCK was associated with increased or decreased autophagy in human umbilical vein endothelial cells, respectively. They report that ROS plays a role in the expression of CLOCK which in turn promotes autophagy and endothelial dysfunction under hypoxia. These results demonstrate a novel mechanism of action of CLOCK in endothelial cells and opens up attractive possibilities of targeting CLOCK for the treatment of endothelial disorders.

Although the existing literature documents role of oxidative stress and autophagy in metabolism and longevity, the growing burden of metabolic disorders and inadequacy of existing therapeutic modalities necessitates further research. Therefore, in this special issue, we aimed to invite investigators to contribute their original research to further our understanding of the role of oxidative stress and autophagy process. We believe that the present issue summarizes some of the current developments in this field and will be of interest to the readers.
